# “Back on Track”: A Mobile App Observational Study Using Apple’s ResearchKit Framework

**DOI:** 10.2196/mhealth.6259

**Published:** 2017-02-28

**Authors:** Martin Zens, Peter Woias, Norbert P Suedkamp, Philipp Niemeyer

**Affiliations:** ^1^ Department of Orthopedic Surgery and Traumatology University Medical Center Freiburg University of Freiburg Freiburg Germany; ^2^ Design of Microsystems Department of Microsystems Engineering (IMTEK) University of Freiburg Freiburg Germany

**Keywords:** mHealth, mobile health, anterior cruciate ligament injury

## Abstract

**Background:**

In March 2015, Apple Inc announced ResearchKit, a novel open-source framework intended to help medical researchers to easily create apps for medical studies. With the announcement of this framework, Apple presented 5 apps built in a beta phase based on this framework.

**Objective:**

The objective of this study was to better understand decision making in patients with acute anterior cruciate ligament (ACL) ruptures. Here, we describe the development of a ResearchKit app for this study.

**Methods:**

A multilanguage observatory study was conducted. At first a suitable research topic, target groups, participating territories, and programming method were carefully identified. The ResearchKit framework was used to program the app. A secure server connection was realized via Secure Sockets Layer. A data storage and security concept separating personal information and study data was proposed. Furthermore, an efficient method to allow multilanguage support and distribute the app in many territories was presented. Ethical implications were considered and taken into account regarding privacy policies.

**Results:**

An app study based on ResearchKit was developed without comprehensive iPhone Operating System (iOS) development experience. The Apple App Store is a major distribution channel causing significant download rates (>1.200/y) without active recruitment. Preliminary data analysis showed moderate dropout rates and a good quality of data. A total of 180 participants were currently enrolled with 107 actively participating and producing 424 completed surveys in 9 out of 24 months.

**Conclusions:**

ResearchKit is an easy-to-use framework and powerful tool to create medical studies. Advantages are the modular built, the extensive reach of iOS devices, and the convenient programming environment.

## Introduction

In March 2015, Apple Inc (Cupertino, CA, USA) announced the launch of ResearchKit, an open-source framework shipped with iPhone Operating System (iOS) 8.3, aiming at revolutionizing medical research studies to its developer community. Medical researchers around the world paid attention to this hot topic addressing their daily challenges [1]. The preview was followed by a 4-week-long waiting time until further documentation, and the framework itself was released to the public [2,3]. The source code is distributed under an open-source license via GitHub (GitHub, Inc) [4].

Immediately after Apple’s announcement, a controversial discussion regarding the impact, significance, and potential risks started in the public and scientific community. Enthusiasts stress how easy it is to recruit participants and that participating becomes as simple as posting on Facebook. Furthermore, it is argued that the data may be more realistic when gathered in daily life instead of an unrealistic laboratory setting. The sensor data provided by the iOS devices will potentially unveil new hypothesis and perspectives on certain diseases. Optimists predict that millions of people will participate in medical studies in the near future only because it becomes simple and accessible. The major advantage of Apple and ResearchKit is its broad market share with millions of potential participants [5]. The main concern of critics is the quality of the gathered information. Missing possibilities to confirm a participant’s illness and challenges in matching sensor data, that is, step count, with physical activity levels are only two objections commonly mentioned [6]. Apart from that, iPhone users are more likely to be higher educated and have a higher income than the average population [7,8]. This bias is another concern often discussed by critics [9]. Our aim was to present a study designed with Apple ResearchKit and provide insights of the development process to other researchers who are interested in this novel framework.

## Methods

### Study Selection

**Figure 1:**
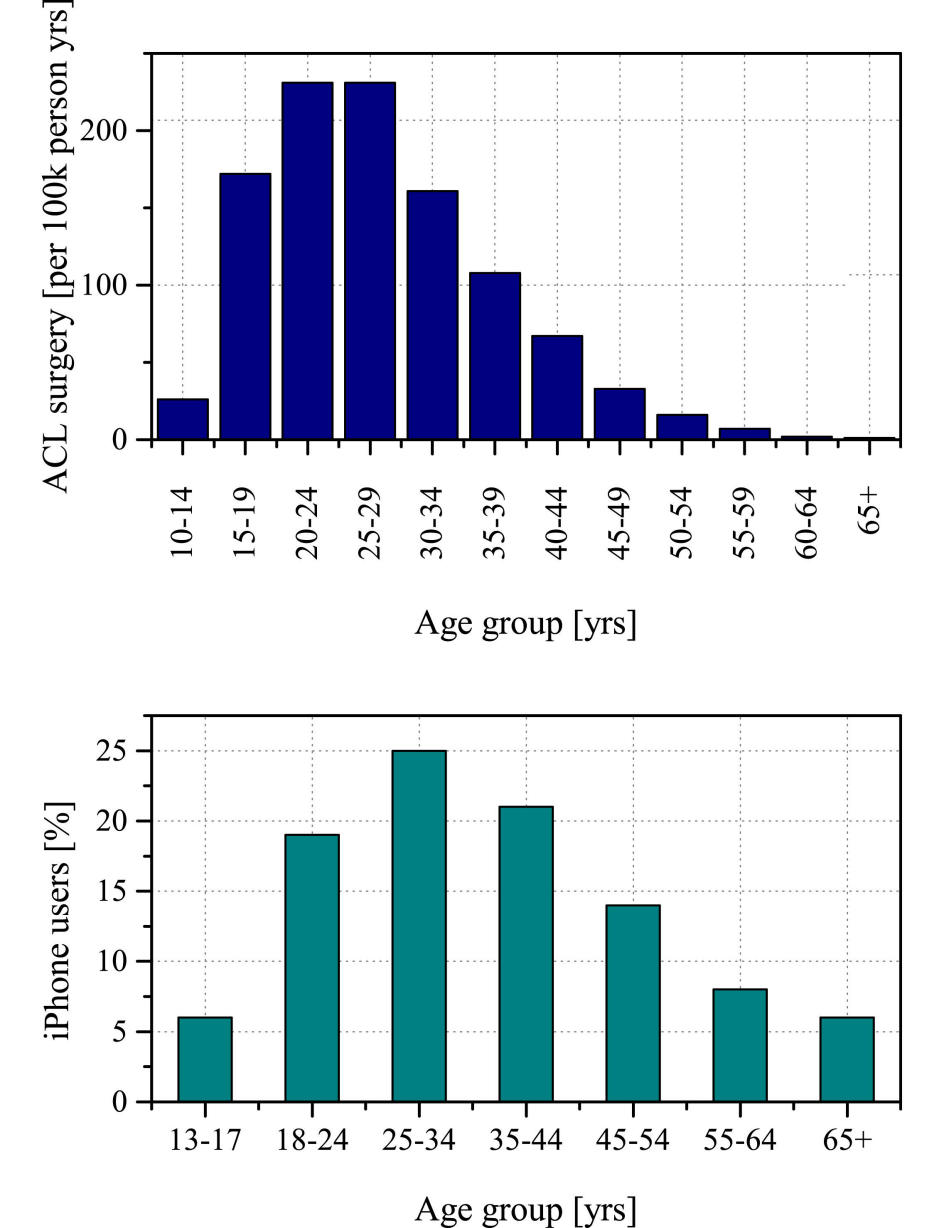
Comparison of age distribution in iPhone users and age of ACL surgery patients taken from a national population-based study in New Zealand.

A research topic to be investigated with Apple ResearchKit was carefully chosen. Due to the preselected group of potential participants and the nature of mobile device studies, the bias of a study depends on the topic [10]. Studies have shown that iPhone users are generally higher educated, have a higher income, and are younger than the average population in developed countries [7]. Ruptures of the anterior cruciate ligament (ACL) occur predominantly in young and active people, and no studies have shown significant correlation between income or education and ACL tears [11-13]. Figure 1 shows the age distribution of iPhone users [14] and ACL [15] surgery in New Zealand. It is concluded that ResearchKit is an appropriate date collection method because the age groups predominantly affected by ACL tears match the predominant age groups of iPhone and potential ResearchKit users. Furthermore, it is proposed that patients are more likely to share information regarding a sports injury via a mobile app than data on psychiatric or severe chronic diseases. Based on these assumptions, a decision-making study evaluating the outcomes of different treatment options for acute ACL tears was performed.

### Study Design

**Figure 2:**
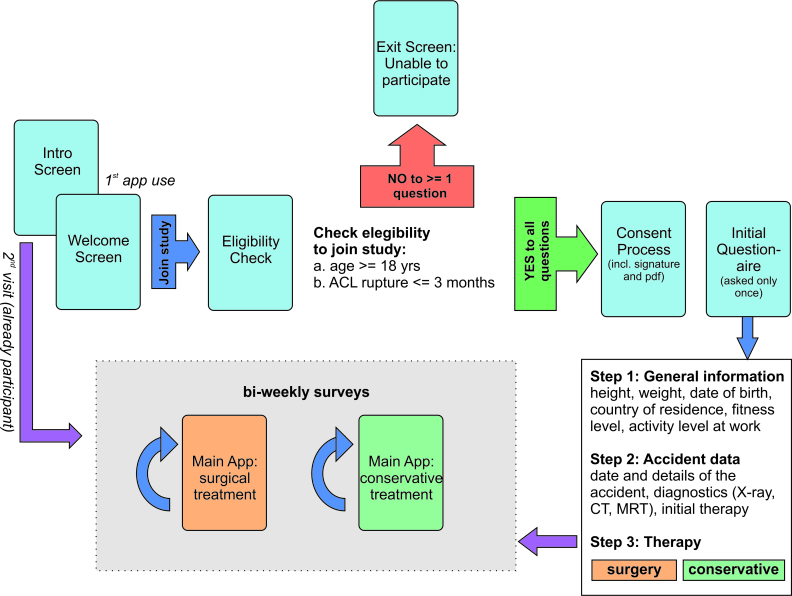
Workflow of the study app depicting the consent process, initial sign-up, and eligibility check.

The study was designed as an observational study collecting data through surveys. The purpose of the study was to evaluate treatment options by capturing patient satisfaction and subjective observations. This allowed the comparison of various treatments based on their outcomes. The app was programmed in 3 languages (German, English, and Spanish) and distributed internationally via the Apple App Stores, thus allowing identification of international differences in ACL therapy. By choosing every country or region with at least one of the apps languages as the official language, it was deployed in 53 regions. Figure 2 presents the workflow of the first mandatory steps when starting the app for the first time. This includes an eligibility check, consenting, and an initial questionnaire.

### Technical Development

#### Modular Concept

The ResearchKit framework provides a modular concept to build research apps. Most apps are distinguishable into 4 sections. Following an intro screen with general information on the study, a consent process is started. Apple ships everything necessary for a digital informed consent in the framework. This step is followed by an initial questionnaire collecting personal information and disease-relevant data, such as diagnostic procedures and previous and current therapies. In case these modules are completed successfully, the participant is registered and may take part in the study through the main app. A main app may be customized according to the investigators’ needs. With ResearchKit, Apple provides easy-to-use modules to create surveys and profiles, access sensor data, and display scores on a dashboard [16]. Well-known features from other apps such as push notifications are also quickly integrated.

**Figure 3:**
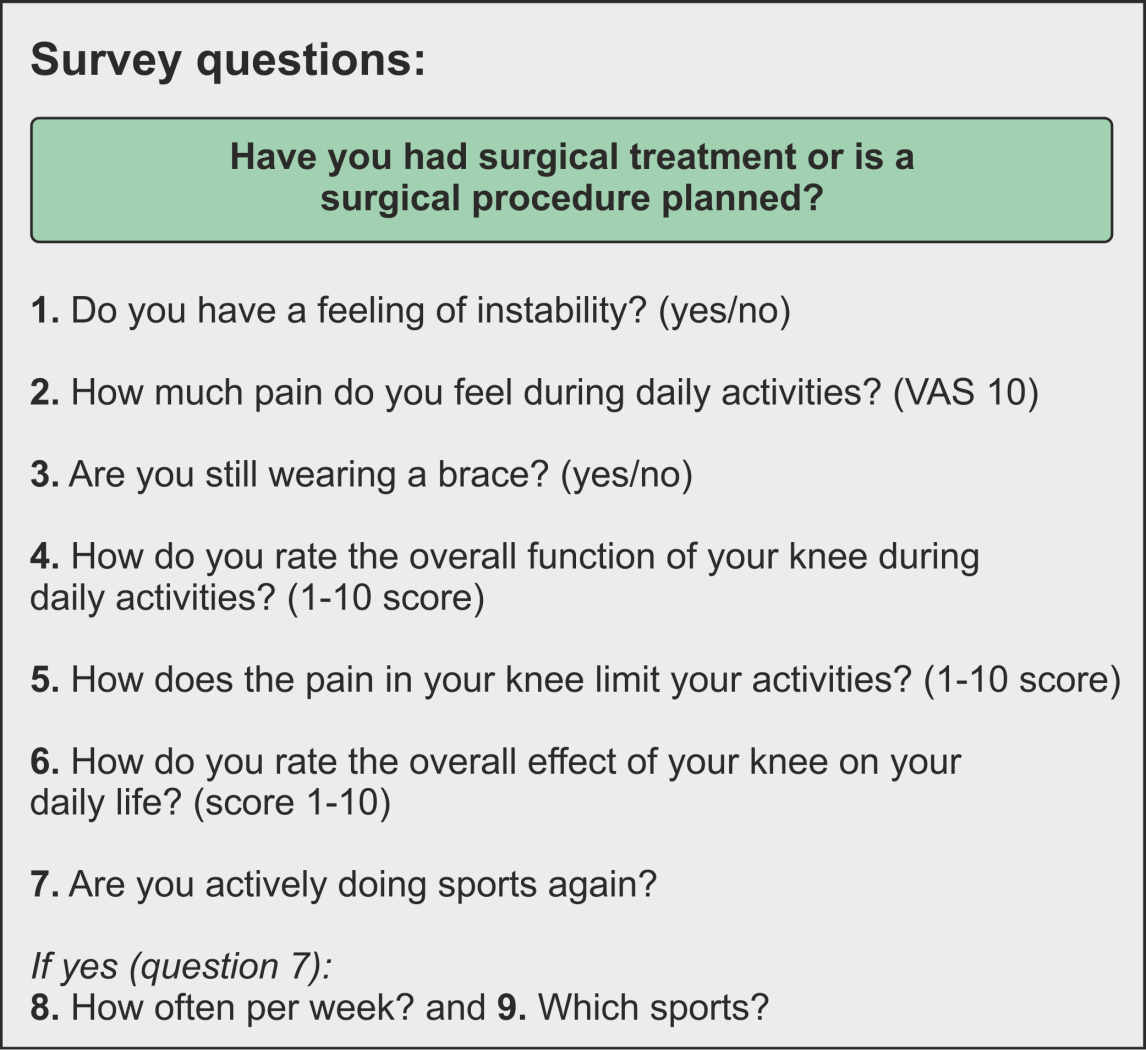
Questions of the bi-weekly survey. The question in the green box was only asked when the participant received a conservative treatment.

The ACL rupture study app presented in this study comprised a survey with 9 questions that have been validated in previous studies. Figure 3 displays these questions. The question in the green box was only asked while a participant received a conservative treatment.

#### Programming Environment

Xcode (Apple Inc, Cupertino, CA, USA) was required to develop iOS apps and thus was also necessary to develop ResearchKit apps. It was distributed free of charge as proprietary software. ResearchKit itself was open source and installed locally for development purposes by cloning the GitHub repository [4].

#### Server Security

**Figure 4:**
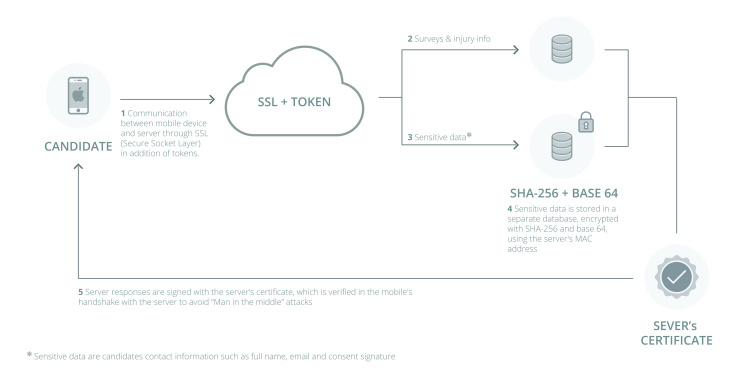
Security concept for data storage and communication.

A server with Ubuntu 14.04 (Canonical Ltd, London, UK) as operating system was set up on the intuitional site to ensure that all data were physically stored in a secure data center in Germany. A Linux, Apache, MySQL, and PHP (LAMP, PHP: Hypertext Preprocessor) system including PHP 5.5 and MySQL 5.5 was installed. All communication between mobile device and institutional server was realized via Secure Sockets Layer (SSL). Different databases were used for sensitive data (name, email, and signature) and study data (surveys and information). Server side encryption was realized using mcrypt_create_iv(), which created a padding vector, padded the serialized value, and encrypted the result using base64 and the padding vector. Afterward it hashed this encrypted value using the media access control (MAC) address of the server. This allowed for the data only to be decrypted on the encryption server. Finally, it encoded with base64 an array containing the JavaScript Object Notation (JSON) encoding of the padding vector, original value, and MAC address. This value was stored in the database. The security concept is depicted in Figure 4.

#### Language Support

Localizable strings were used in this study to realize multilanguage support for the ACL rupture app. ResearchKit supported this commonly used technique in iOS development. Strings were placed in separate language files with a unique identifier used to place the strings at the appropriate location in the app. Initially, the app was designed in English. By using the concept of localizable strings, language support for Spanish and German was added by duplicating the English language file and adapting it for the other 2 languages. Changes to the graphical user interface and design were only necessary when adding language support for a language that was not based on the Latin or similar typeset, that is, Chinese or Japanese. Hunt et al [17] showed that language barriers had a significant impact on clinical studies. To overcome these barriers it was necessary to provide a multilanguage version of an app to collect reliable data internationally.

#### Ethics and Data Protection

This study was approved by the local ethics committee and registered with the German Clinical Trials Register (DRKS-ID: DRKS00009270). The approval was mainly based on the thorough implementation of German data protection laws. Due to the fact that the app server, all data, and the study center were based in Germany, only German data protection laws and retention policies applied for the app, although study participants were located worldwide. Supervision was carried out by German local and federal authorities.

## Results

A first ResearchKit app to investigate the decision-making process in the treatment of ACL tears was developed. The app had multilanguage support and was distributed through Apple App Stores. It was added to the portfolio of all App Stores in regions and countries with German, English, or Spanish as the official language, thus resulting in 53 regional stores.

**Figure 5:**
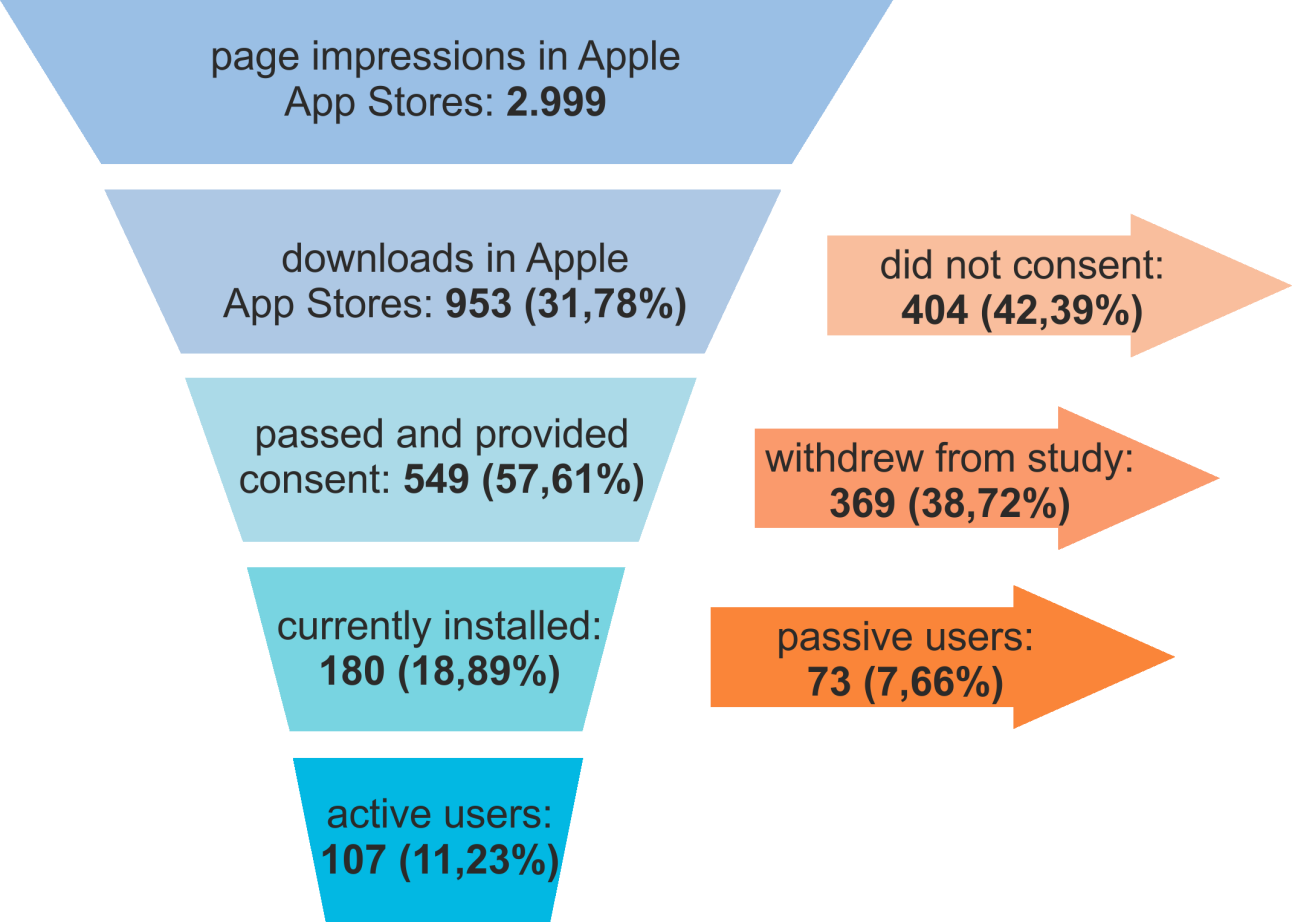
Composition and evolution of study participants.

Within 9 months of recruitment, the App Store website was accessed 2999 times and the app was downloaded 953 times (31.8% conversion rate, 953/2999). A total of 549 participants (57.6% of all downloads, 549/953) completed the consent form successfully and joined the study. Currently, the app is installed on 180 iOS devices (18.9%, 180/953) and 107 participants (11.2%, 107/953) are actively participating in study activities at the moment. A participant is considered to be actively participating when still enrolled in the study and having completed the last 2 surveys. Figure 5 depicts the composition and evolution of participants. In this ongoing study, 424 surveys were completed within a total duration of 9 months (1.57 per day).

Preliminary data show downloads from 21 of 53 regions with a majority of these originating from Germany, Austria, and Switzerland (85%, 91/107). A gender analysis of all participants showed a ratio of 74:26 in favor of the male sex. The predominant age group was 25-34 years (54%, 58/107). No participants older than 56 years were registered for the study.

## Discussion

The idea of conducting medical studies via the Internet and by using mobile devices is not new [18-21]. Previously, this technique was used to improve communication, data collection, and data quality in studies with participants known personally to the investigators, that is, somehow they are affiliated with the study center. A major advantage and novelty of ResearchKit is the ability to conduct studies and recruit participants unrelated to the study center. Furthermore, ResearchKit simplifies the process of developing such apps and thus makes this technology affordable for research groups around the world.

Mobile apps allow for groups of people to participate in medical studies, which are underrepresented in conventional studies, such as young and active people or those living in remote locations [16]. Researchers also hope to address patients suffering from mental illnesses with new mobile study apps. This specific target group has been difficult to reach with conventional studies [22].

Opening up mobile app development to a large community and conducting large-scale studies for thousands of participants imply the necessity for quality assurance on both sides. The quality of medical mobile apps has been discussed for several years [23]. Recently, the Food and Drug Administration announced to implement stricter regulations for medical apps. Although this announcement targeted apps, it might possibly harm patients through misleading, unfiltered, or incorrect information; the quality of study apps might also decrease with the rapidly increasing number. This issue needs to be addressed by independent institutions that check and certify mobile study apps [24]. In addition, ethics committees have to be educated on the new technologies and the implications of an increasing number of studies conducted via mobile devices. The quality of ResearchKit apps may benefit from the fact that the framework is open source. Researchers who are new to the field can avoid pitfalls by using a well-tested platform with many contributors.

According to the National Health Service (NHS) study in the United Kingdom, development costs currently range from £1000 to £30,000 depending on the extent and functionality of the desired app [16,25,26]. The modular design concept of ResearchKit reduces the initial development cost of mobile apps, as well as the following maintenance cost. Furthermore, the cost per participant of a mobile app study is significantly lower compared with that of conventional studies [27].

Moreover, the acceptance of mobile health care studies in the general public may possibly increase with Apple’s involvement in this sector. According to a recent study, medical students and junior doctors are very enthusiastic about mobile health (mHealth) solutions. Future development in this sector may very likely be driven by this peer group [28].

In the last decade, browser-based Internet surveys were the most common technology used for study surveys in the United States [29]. This trend will shift toward mobile devices in the near future. A thorough understanding of this technology, with its risks and benefits, is needed by all parties involved in the process. This includes ethics committees, data protection officers, data storage and security specialists, app developers, public relations officers, medical researchers, and participants.

Recruitment and retention of participants may also change in contrast to browser-based surveys. All traditional recruitment methods that are currently used to win participants for Web-based survey studies, that is, Google AdWords, Facebook ads, social media and forum posts, newspaper ads, TV, radio, flyers, and doctors, can also be used for mobile phone–based study apps. However, participant retention is low with browser-based Web-based surveys [30]. This may improve with mobile phone–based study apps. Especially, the push-notification function of iOS devices, which is easily implemented in ResearchKit apps, allows the investigators to send notifications, reminders, and information directly on a participant’s mobile device.

Apart from that, this study revealed that passive recruitment was insufficient to enroll large numbers of participants. New recruitment channels have to be tapped. Solely relying on passive recruitment—meaning active participants who might find the study app through search engines—does not allow conducting large big data studies. Furthermore, keeping up the motivation to participate is challenging. Possible solutions are an instant feedback of study results, a gamification approach, or providing relevant treatment information.

### Comparison With Prior Work

Only very few prior scientific studies have been published explicitly reporting on experience and first steps with Apple ResearchKit. Mobile-device–based health care studies are numerous [21,24,31]. Data have to be gathered and analyzed in order to compare the ResearchKit studies with different methods and techniques.

The mPower study was created and launched within the beta phase of Apple ResearchKit. Bot et al recently published preliminary results [32]. The percentage of consenting participants (34.5%) was reported to be significantly lower than that determined in this study (57.6%, 549/953). The resulting percentages of active participants, however, are comparable between both studies, with 10.9% in the mPower study and 11.2% (107/953) in this study.

### Conclusions

Apple ResearchKit provides an interesting tool to easily create and distribute medical research apps that are simple to access and use for the public. The concept of a major company developing and promoting software, which targets this issue, is new. Many questions regarding this novel technology remain unanswered. Further investigations, especially regarding bias and public acceptance, need to be performed. Data quality and validity have to be evaluated in the further course in order to derive reliable conclusions for future mHealth developments. In addition, novel approaches to acquire participants and preserve the initial motivation to participate have to be found in order to reduce dropout rates.

## Abbreviations

ACL: anterior cruciate ligament
iOS: iPhone Operating System
LAMP: Linux, Apache, MySQL, PHP
PHP: Hypertext Preprocessor
SSL: Secure Sockets Layer
